# Lung cancer incidence differences in migrant men in Belgium, 2004–2013: histology-specific analyses

**DOI:** 10.1186/s12885-021-08038-6

**Published:** 2021-03-30

**Authors:** Katrien Vanthomme, Michael Rosskamp, Harlinde De Schutter, Hadewijch Vandenheede

**Affiliations:** 1 Interface Demography, Department of Social Research, Faculty of Economic and Social Sciences & Solvay Business School, Vrije Universiteit Brussel, Pleinlaan 2, 1050 Brussels, Belgium; 2 Research Department, Belgian Cancer Registry, Koningsstraat 215, 1210 Brussels, Belgium

**Keywords:** Belgium, Immigrants, Lung Cancer, Incidence, Inequalities, Histology-specific

## Abstract

**Background:**

Immigrants make up an important share of European populations which has led to a growing interest in research on migrants’ health. Many studies have assessed migrants’ cancer mortality patterns, yet few have studied incidence differences. This paper will probe into histology-specific lung cancer incidence by migrant origin aiming to enhance the knowledge on lung cancer aetiology and different risk patterns among population groups.

**Methods:**

We used data on all lung cancer diagnoses during 2004–2013 delivered by the Belgian Cancer Registry individually linked with the 2001 Belgian Census and the Crossroads Bank for Social Security. Absolute and relative inequalities in overall and histology-specific lung cancer incidence have been calculated for first-generation Italian, Turkish and Moroccan migrant men aged 50–74 years compared to native Belgian men.

**Results:**

Moroccan men seemed to be the most advantaged group. Both in absolute and relative terms they consistently had lower overall and histology-specific lung cancer incidence rates compared with native Belgian men, albeit less clear for adenocarcinoma. Turkish men only showed lower overall lung cancer incidence when adjusting for education. On the contrary, Italian men had higher incidence for overall lung cancer and squamous cell carcinoma, which was explained by adjusting for education.

**Conclusions:**

Smoking habits are likely to explain the results for Moroccan men who had lower incidence for smoking-related histologies. The full aetiology for adenocarcinoma is still unknown, yet the higher incidence among Italian men could point to differences in occupational exposures, e.g. to carcinogenic radon while working in the mines.

## Background

### Background of the study

Immigrants make up an important share of the European populations nowadays [1]. This has led to a growing interest in research on the health of immigrants. Several studies on migrant mortality have observed a migrant mortality advantage (MMA) [2]. Despite their often more disadvantaged living situation, migrants tend to have lower mortality compared with the host population. This advantage is especially pronounced for lifestyle-related diseases whereas migrants’ mortality from infectious diseases tends to be higher than that of the native population [1, 3, 4]. Moreover, this mortality advantage is in particular evident for migrants moving from less- to more-industrialized countries [5]. In addition, with increasing length of stay in the host country, the mortality patterns of migrants seem to converge towards those of the host population as they are exposed to physical, social and environmental influences in the host country [1–3, 5–7]. Mortality can be considered as the ‘ultimate inequality outcome’ [8]. Yet it often remains unclear whether the observed patterns reflect inequalities in incidence or in case-fatality (survival) [9]. Therefore, it is particularly interesting to study differences in disease occurrence and survival to see whether this migrant advantage holds also for these health outcomes. Studies on migrants’ incidence, survival and mortality can produce additional knowledge on disease aetiology which is important for prevention and treatment programs, and to identify migrants’ health care needs [1, 3, 4, 10–16]. As migrants have been exposed to multiple environments before, during and after migration, they are a particularly apt group to study health differences in order to reveal novel insights on the causes of diseases [1, 15, 17–19].

Belgium is a particularly suitable setting to analyse migrant health differences, as it has a long history of migration [12, 20, 21]. In the 1950s and 1960s, Belgium was in strong need of labour migrants to overcome the labour shortages in certain heavy industries such as mining [20, 21]. As a result of this labour migration, large groups of especially Southern European, Turkish and Moroccan men immigrated to Belgium, later permanently settling as their wives followed. These labour migrants generally find themselves in the lower socioeconomic strata [3, 21, 22]. Nowadays the traditional first-generation (FG) labour migrants have reached the older age groups, making their health status and health care issues an increasingly important aspect to follow-up [15]. It is necessary to document the health status of these FG migrants as they make up an important part of the population and as they may face different health risks [22]. Furthermore, disease-specific analyses are essential to disentangle the various mechanisms at play, be it lifestyle, genetics, environmental exposure or access to and quality of health care [12]. In addition to different health risks and exposures, immigrants’ individual socioeconomic and sociodemographic circumstances also play a role in health-related behaviour and disease risk [5, 23, 24].

In this paper we focus on lung cancer among men, as it is the second most common cancer and the most common cause of cancer death in Belgium and Europe among men [25, 26]. As lung cancer is especially prevalent in high-income countries, previous reports on lung cancer mortality by migrant group have observed a MMA in several European countries [10, 27] as well as in Belgium [11]. Among the traditional labour migrant groups in Belgium, the MMA for lung cancer has been especially confirmed for Turkish and Moroccan men [11]. Lung cancer is known to be a highly fatal disease, with 5-years relative survival proportions of about 18% among Belgian men [25]. Considering this, we may expect a migrant advantage for lung cancer incidence as well. However, since we focus on FG migrants who immigrated to Belgium decades ago, we can expect a certain degree of convergence towards the host country’s disease patterns due to acculturation [4, 13, 28–31]. Previous research on migrant differences in lung cancer in Norway for example have shown increasing lung cancer incidence rates among migrants converging to the host country’s levels [30].

In this study we dig deeper than previous studies as we probe into lung cancer incidence patterns by migrant group as well as by histological subtype. Lung cancer appears in several histological subtypes, for which the risk factors are somewhat different [32–38]. The dominant role of cigarette smoking in lung cancer occurrence has been well established [32–36, 39, 40]. Cigarette smoking is associated with all main histological types of lung cancer, although the strength of the association differs by subtype: the association is strongest for small-cell lung carcinoma (SCLC) and squamous cell carcinoma (SCC) and less strong for adenocarcinoma (ADC) [32–36]. In addition, ADC is the most common lung cancer type among non-smokers, which suggest that there are other (still unknown) factors involved in ADC aetiology than smoking [32, 36, 40, 41]. Studying migrant lung cancer incidence patterns by histological subtypes enhances the knowledge on the aetiology of lung cancer and on the different risk patterns among population groups.

### Study aims

This study is the first one to document lung cancer incidence rates in males during 2004–2013 by migrant group (as measured by country of origin) and histological subtype. We look into three research questions: First, do overall and histology-specific lung cancer incidence rates among male Belgian residents differ by migrant group as compared to native Belgian men? Second, are there differences in tumour characteristics between migrant groups and native Belgian men, taking the histological subtype into account? For instance, are there differences in stage at diagnosis, which is an important prognostic factor [42]. Third, does accounting for sociodemographic and socioeconomic variables alter the association between country of birth and histology-specific lung cancer incidence? Immigrants are often in the lower socioeconomic strata, but are deprived migrants equally affected by their socioeconomic position than deprived natives [43]?

## Methods

### Dataset and study population

This population-based study used individually-linked data from three administrative sources: (i) the Belgian Cancer Registry including all lung cancer diagnoses between 2004 and 2013, together with key information on the tumour histology and stage at diagnosis; (ii) the Belgian census of October 1st 2001 containing socioeconomic and sociodemographic information (i.e. age, gender, migrant origin, civil status, educational attainment, home ownership and region of residence); (iii) the Crossroads Bank for Social Security (CBSS) with data on mortality and emigration until December 31st 2013. The linkage of the three data sources was performed by a trusted third party (e-Health) delivering the merged dataset in a pseudonymized format.

For this study we selected FG migrant men from the traditional labour migrant groups in Belgium. We choose this group as they undertook the migration journey and therefore experienced exposures in different settings while at the same time, they have been residing long enough in Belgium to have adopted the natives’ lifestyle. Migrant group was based on the individual’s nationality at birth (available in the census) to define their country of origin. If this information was missing, current nationality was used. In this study we included the three largest groups of traditional labour migrants, i.e. migrant men from Italian, Turkish and Moroccan descent [20], and compared them with Belgian men without a migration background (from now on referred to as native Belgian men). To catch the group of FG traditional labour migrants, we selected only migrants who have been residing more than 10 years in Belgium at the moment of the census.

A retrospective register-based cohort study was conducted based on all Belgian men and FG Italian, Turkish and Moroccan migrant men residing in Belgium for more than 10 years, aged between 50 to 74 at the start of the follow-up period (January 1st, 2004). This population cohort was followed until one of the following events occurred: lung cancer diagnosis, emigration, death, reaching the age of 75 years or end of follow-up (December 31st, 2013). This age group was chosen to ensure to have a sufficient number of lung cancer cases (lower age limit) as well as a sufficient number of immigrant men (upper age limit).

### Variables

The outcome event in this study was being diagnosed with lung cancer during the follow-up period of 2004–2013 according to the International Classification of Diseases (ICD-10) code C34. If patients had multiple tumours within the study period, only the first one was taken into account. All lung cancer diagnoses during the follow-up period were considered for the incidence analyses. Making use of the data of the Belgian Cancer Registry, we were able to classify the lung cancer diagnoses by histological subtype. We thus studied not only overall lung cancer but also small-cell lung carcinoma (SCLC); three types of non-small-cell lung carcinoma, i.e. squamous cell carcinoma (SCC), adenocarcinoma (ADC) and large-cell lung undifferentiated carcinoma (LCLC); and ‘other’ histology containing other and unspecified lung cancers. The corresponding ICD-O-3 codes for the subtypes based on histology are listed in Appendix. Next to histological subtype, we disposed of information on the stage at diagnosis, which is a combination of tumour, nodes and metastasis (known as TNM-stage, classified according to the applicable versions for the studied period) [44]. Staging information relied on combined stage, which is a combination of clinical and pathological stage prioritizing the latter, except in case of distant metastases which were always considered stage IV. Since the number of lung cancer cases were rather limited by migrant group and histological subtype, we decided to distinguish early stage (stages I-II) from late stage cancers (stages III-IV). Patients for whom the stage at diagnosis was missing were reported separately.

In this study we aimed to assess whether migrant origin was associated with lung cancer incidence, as well as the role of different sociodemographic and socioeconomic variables in this regard. Two sociodemographic variables were included: civil status and region of residence. ‘Civil status’ was operationalized as being married, divorced, single or widowed and measured at the 2001 census. ‘Region of residence’ at the 2001 census was also adjusted for, comprising Flanders, Wallonia and the Brussels-Capital Region (BCR). Additionally, to take into account individuals' socioeconomic reality during different life stages [9], two indicators of socioeconomic position (SEP) were included: educational attainment and home ownership. Educational attainment reflects chances early in life and is related to job chances later in life. However, it may not be the best suitable indicator for FG immigrants [45, 46] and therefore home ownership, which refers to the economic assets at the household level was also included. ‘Educational attainment’ was operationalized according to the International Standard Classification of Education (ISCED): primary education or no diploma (ISCED 0–1), lower secondary education (ISCED 2), upper secondary education (ISCED 3–4), and tertiary education (ISCED 5–6). Home ownership differentiated home owners from tenants of a dwelling. Missing categories were treated as separate category for all variables as this might be not randomly distributed according to migrant origin. For educational attainment for instance, the percentage of missing values was larger among migrants than native men, which could be due to the fact that education outside Belgium had not been registered [45].

### Statistical analyses

Both absolute and relative measures of overall and histology-specific lung cancer incidence inequalities by migrant origin were assessed. The person-time at risk by 5-year age groups was calculated for each person in the study cohort between 2004 and 2013 in order to calculate truncated (50–74 years) age-standardized lung cancer incidence rates (ASRs) and 95% confidence intervals (CI) by histological subtype and migrant group. We considered the age distribution of the total population at the start of the follow-up period (January 1st, 2004) as the standard population. The weights in the age range under study equalled the age-specific reference population numbers divided by the total reference population between the ages of 50 and 75. Rates were calculated for all groups. For all histology-specific lung cancers, mean age at diagnosis and combined TNM stage at diagnosis were calculated for each migrant group.

In addition, we calculated relative lung cancer incidence rate ratios (IRR) and 95% CI by migrant group, using a Poisson distribution with the log of the person-time as offset. These relative inequalities were calculated for all histological subtypes of lung cancer as well as overall lung cancer, comparing the cancer incidence rates of migrant men with those of native Belgian men. All relative models were adjusted for age at the start of the follow-up on 01/01/2004, which was included as a continuous variable. The sociodemographic and socioeconomic variables have been added separately to the additional models. Model 1 was adjusted for age only; Model 2 for age and civil status; Model 3 for age and educational attainment; Model 4 for age and home ownership; Model 5 for age and region at census; and Model 6 was adjusted for all variables of interest. All analyses have been performed using SAS 9.3 (SAS Institute Inc.).

## Results

### Description of the study population

The study population contained all Belgian and FG Italian, Turkish and Moroccan men aged 50 to 74 years at the start of the follow-up, as described in Table 1. Turkish and Moroccan men were on average a bit younger than their Belgian and Italian counterparts and had on average arrived about 6 to 7 years later in Belgium than the Italian migrants. The majority of Italian migrants lived in Wallonia, Turkish in Flanders and Moroccan in the Brussels-Capital Region. Among all groups, the majority was married, although with lower percentages among Belgian and Italian men. Migrant men were much often lower educated compared with their Belgian counterparts. Also, the percentage of missing values on this variable was much higher among migrants. Finally, the majority of men in all groups were owners of a dwelling, although the percentage was lower among Turkish and Moroccan men.

**Table 1:** Description of the study population aged 50 to 74 years at the start of the follow-up (01/01/2004), by country of origin, Belgium

	Belgian	Italian	Turkish	Moroccan
Number of persons	1,791,275	45,572	13,901	25,880
Mean age at start follow-up	55.31	57.03	52.13	53.65
Mean years since immigration	–	33.76	26.23	26.91
Region of residence (%)				
Flanders	65.46	13.33	47.97	29.84
Brussels-Capital Region	5.18	10.72	25.57	51.73
Wallonia	29.36	75.95	26.45	18.44
Civil status (%)				
Married	75.98	82.72	95.01	91.26
Single	10.61	6.64	1.40	3.28
Divorced	10.78	8.24	2.86	4.93
Widow	2.63	2.40	0.73	0.53
Educational attainment (%)				
Primary or less	16.74	28.12	31.56	13.96
Lower secondary	26.61	25.07	17.30	15.39
Upper secondary	23.66	12.86	10.22	11.59
Tertiary	22.65	5.46	4.42	7.98
Missing	10.34	28.48	36.50	51.08
Home ownership (%)				
Owner	79.09	77.55	67.51	54.46
Tenant	16.74	17.30	23.19	36.44
Missing	4.17	5.15	9.30	9.10

The number of persons, mean age and mean years since migration were measured at the start of the follow-up (01/01/2004). Information of region of residence, civil status, educational attainment and home ownership was measured at the census (01/10/2001)

### Description of the lung cancer cases and absolute incidence inequalities by histological subtype and migrant group in Belgium

In this paper we included lung cancers diagnosed between 2004 and 2013 in the male study population. We performed the analyses for overall lung cancer as well as histology-specific. ADC was the most common histological subtype, closely followed by SCC, whereas LCLC was the least common lung cancer subtype (Table 2). Only among men from Turkish descent, the incidence rates for SCC and ADC were similar.

**Table 2:** Number of cases, person-years and truncated age-standardized incidence rates (ASR) with 95% confidence intervals (95% C.I.) per 100,000 person-years among the study population aged 50 to 74 years, by histological subtype and country of origin, Belgium, 2004–2013

		Nr. of cases	Person-years	ASR (95% C.I.)
**Overall lung cancer**	Belgian	30,008	11,922,223	253.8 (251.0–256.7)
	Italian	937	335,372	270.9 (253.3–288.5)
	Turkish	199	84,828	271.9 (233.6–310.3)
	Moroccan	333	171,470	199.1 (177.6–220.5)
**Small-cell lung carcinoma**	Belgian	4818	11,922,223	40.7 (39.6–41.9)
	Italian	129	335,372	37.5 (30.9–44.1)
	Turkish	37	84,828	51.6 (34.7–68.4)
	Moroccan	49	171,470	29.4 (21.2–37.7)
**Squamous cell carcinoma**	Belgian	9361	11,922,223	79.5 (77.8–81.1)
	Italian	303	335,372	88.3 (78.2–98.4)
	Turkish	68	84,828	91.8 (69.6–113.9)
	Moroccan	87	171,470	53.2 (42.0–64.4)
**Adenocarcinoma**	Belgian	10,829	11,922,223	91.2 (89.5–92.9)
	Italian	392	335,372	112.8 (101.4–124.1)
	Turkish	67	84,828	90.1 (68.2–112.0)
	Moroccan	157	171,470	92.9 (78.3–107.4)
**Large-cell undifferentiated carcinoma**	Belgian	1485	11,922,223	12.6 (12.0–13.2)
	Italian	26	335,372	7.5 (4.6–10.4)
	Turkish	*N* < 10	84,828	8.6 (1.6–15.5)
	Moroccan	*N* < 15	171,470	7.5 (3.4–11.6)
**Other/unknown**	Belgian	3515	11,922,223	29.9 (28.9–30.9)
	Italian	87	335,372	24.9 (19.6–30.2)
	Turkish	< 25	84,828	29.9 (17.0–42.8)
	Moroccan	27	171,470	16.1 (10.0–22.2)

In absolute terms, some differences by migrant origin were observed (Table 2). Migrant men from Moroccan descent had a significant lower overall lung cancer truncated ASR compared with all other groups. To illustrate, about 200 Moroccan men per 100,000 person-years were diagnosed with lung cancer during the study period (95% CI: 177.6–220.5) compared with about 250 Belgian men (95% CI: 251.0–256.7). This advantage for Moroccan men compared with native Belgian men was significant for all histological subtypes, except for ADC. For SCLC, migrant men from Moroccan descent had lower incidence rates compared with native Belgian men: the latter had an ASR of 40.7 per 100,000 person-years (95% CI: 39.6–41.9) whereas migrant men from Moroccan descent had an ASR of 29.4 (95% CI: 21.2–37.7). In addition, for SCC, Moroccan men had lower incidence rates compared to all other origin groups: e.g. native Belgian men had an ASR of 79.5 per 100,000 person-years (95% CI: 77.8–81.1) whereas migrant men from Moroccan descent had an ASR of 53.2 (95% CI: 42.0–64.4). Similar to Moroccan men, Italian immigrant men had favourable LCLC incidence rates compared with native Belgians with 7.5 new diagnoses per 100,000 person-years among Italians (95% CI: 4.6–10.4) versus 12.6 among native Belgian men (95% CI: 12.0–13.2). Finally, also lung cancer incidence rates with unknown or other histology were lower among Moroccan men as compared to Native Belgian men.

The only exception to this advantageous incidence pattern among Moroccan men was ADC for which there was no difference with native Belgian men. Inversely, among men from Italian descent, ADC incidence was significantly higher compared with native Belgians with 112.8 Italian men newly diagnosed per 100,000 person-years (95% CI: 101.4–124.1) compared with 91.2 among Belgian men without a migration background (95% CI: 89.5–92.9).

Table 3 describes the mean age at diagnosis and the TNM stage of the newly diagnosed lung cancer cases. Turkish and Moroccan men in the studied age group were on average a bit younger at lung cancer diagnosis. Stage at diagnosis for overall lung cancer incidence was generally most favourable among Turkish migrant men, whereas the percentage of missing TNM information was highest among Italian migrants. Moreover, the results showed that SCC (for all origin groups) and ADC (in particular for Turkish and Moroccan men) were most often diagnosed at earlier stages. In addition, we observed fluctuations in stage at diagnosis across migrant group as well. For instance, Turkish men were more often diagnosed in advanced stages for SCC compared with the other men, whereas this was the case for native Belgian and Moroccan men for SCLC (the latter not statistically significant though).

**Table 3:** Description of lung cancer cases among the study population aged 50 to 74 years, by histological subtype and country of origin, Belgium, 2004–2013

	Belgian	Italian	Turkish	Moroccan
**Overall lung cancer**				
Mean age at diagnosis	67.35	67.62	65.71	65.40
Combined TNM stage (%)				
I-II	21.53	22.63	26.50	19.94
III-IV	57.35	48.24	54.00	58.33
Missing	21.12	29.14	19.50	21.73
**Small-cell lung carcinoma**				
Mean age at diagnosis	64.84	64.35	65.51	63.92
Combined TNM stage (%)				
I-II	4.71	5.43	5.41	4.08
III-IV	61.58	49.61	51.35	61.22
Missing	33.71	44.96	43.24	34.69
**Squamous cell carcinoma**				
Mean age at diagnosis	65.81	65.83	63.94	65.83
Combined TNM stage (%)				
I-II	30.44	28.38	27.94	25.00
III-IV	50.91	45.21	61.76	51.14
Missing	18.65	26.4	10.29	23.86
**Adenocarcinoma**				
Mean age at diagnosis	64.33	65.13	63.54	62.59
Combined TNM stage (%)				
I-II	22.17	26.79	36.76	23.42
III-IV	61.12	50.26	45.59	58.86
Missing	16.71	22.96	17.65	17.72
**Large-cell undifferentiated carcinoma**				
Mean age at diagnosis	65.26	64.85	65.79	59.12
Combined TNM stage (%)				
I-II	11.85	11.54	N < 10	N < 15
III-IV	63.37	50.00	N < 10	N < 15
Missing	24.78	38.46	N < 10	N < 15
**Other/unknown**				
Mean age at diagnosis	65.72	65.24	66.21	65.81
Combined TNM stage (%)				
I-II	23.02	12.64	33.33	14.81
III-IV	54.52	47.13	52.38	66.67
Missing	22.45	40.23	14.29	18.52

### Relative lung cancer incidence inequalities by histological subtype and migrant group in Belgium

The results from the relative incidence inequalities models were somewhat different by histological subtype, therefore we will discuss the results by origin group (Table 4). For all histological subtypes, six statistical models were performed: from only age-adjusted (model 1) to fully adjusted for age, sociodemographic and socioeconomic variables (model 6).

**Table 4:** Incidence rate ratios (with 95% confidence interval) by histological subtype and country of origin, adjusted for age and sociodemographic and socioeconomic indicators - Belgian male residents aged 50–74 years, 2004–2013

**Overall lung cancer**	**Model 1**	**Model 2**	**Model 3**	**Model 4**	**Model 5**	**Model 6**
Migrant origin						
Belgian	(ref.)	(ref.)	(ref.)	(ref.)	(ref.)	(ref.)
Italian	1.08 (1.01–1.15)	1.09 (1.02–1.16)	0.90 (0.84–0.96)	1.07 (1.00–1.14)	0.99 (0.92–1.05)	0.85 (0.80–0.91)
Turkish	1.05 (0.91–1.21)	1.10 (0.96–1.26)	0.82 (0.71–0.94)	0.97 (0.84–1.11)	1.05 (0.91–1.21)	0.82 (0.71–0.95)
Moroccan	0.81 (0.72–0.90)	0.84 (0.75–0.93)	0.63 (0.57–0.71)	0.70 (0.63–0.78)	0.81 (0.73–0.91)	0.63 (0.56–0.70)
Age	1.07 (1.07–1.07)	1.07 (1.07–1.07)	1.06 (1.06–1.06)	1.07 (1.07–1.07)	1.07 (1.07–1.07)	1.07 (1.06–1.07)
Civil status						
Single		1.11 (1.06–1.15)				0.95 (0.91–0.99)
Divorced		1.43 (1.39–1.48)				1.19 (1.15–1.23)
Widow		1.21 (1.14–1.28)				1.09 (1.03–1.16)
Married		(ref.)				(ref.)
Educational attainment						
Primary or less			2.44 (2.34–2.53)			2.32 (2.22–2.41)
Lower secondary			1.93 (1.85–2.00)			1.86 (1.79–1.93)
Upper secondary			1.52 (1.45–1.58)			1.49 (1.43–1.56)
Tertiary			(ref.)			(ref.)
Missing			2.52 (2.41–2.63)			2.26 (2.16–2.36)
Home ownership						
Tenant				1.72 (1.67–1.76)		1.54 (1.50–1.59)
Owner				(ref.)		(ref.)
Missing				1.56 (1.48–1.64)		1.30 (1.23–1.37)
Region of residence						
Flanders					(ref.)	(ref.)
Brussels-Capital Region					1.03 (0.98–1.08)	0.98 (0.93–1.03)
Wallonia					1.20 (1.17–1.23)	1.19 (1.17–1.22)
**Small-cell lung carcinoma**	**Model 1**	**Model 2**	**Model 3**	**Model 4**	**Model 5**	**Model 6**
Migrant origin						
Belgian	(ref.)	(ref.)	(ref.)	(ref.)	(ref.)	(ref.)
Italian	0.92 (0.78–1.10)	0.93 (0.78–1.11)	0.75 (0.63–0.90)	0.92 (0.77–1.09)	0.84 (0.70–1.00)	0.70 (0.58–0.83)
Turkish	1.20 (0.87–1.66)	1.25 (0.91–1.73)	0.91 (0.65–1.25)	1.12 (0.81–1.55)	1.21 (0.88–1.68)	0.91 (0.66–1.26)
Moroccan	0.73 (0.55–0.97)	0.75 (0.57–1.00)	0.56 (0.42–0.74)	0.64 (0.48–0.85)	0.75 (0.56–1.00)	0.55 (0.41–0.74)
Age	1.07 (1.06–1.07)	1.07 (1.07–1.07)	1.06 (1.05–1.06)	1.07 (1.07–1.07)	1.07 (1.07–1.07)	1.06 (1.06–1.06)
Civil status						
Single		1.08 (0.97–1.20)				0.93 (0.83–1.03)
Divorced		1.40 (1.28–1.52)				1.17 (1.07–1.28)
Widow		1.15 (0.99–1.34)				1.03 (0.89–1.20)
Married		(ref.)				(ref.)
Educational attainment						
Primary or less			3.03 (2.73–3.37)			2.92 (2.62–3.24)
Lower secondary			2.32 (2.09–2.57)			2.24 (2.02–2.49)
Upper secondary			1.75 (1.57–1.96)			1.73 (1.55–1.94)
Tertiary			(ref.)			(ref.)
Missing			3.08 (2.75–3.45)			2.84 (2.53–3.19)
Home ownership						
Tenant				1.68 (1.57–1.80)		1.49 (1.39–1.60)
Owner				(ref.)		(ref.)
Missing				1.39 (1.22–1.59)		1.13 (0.98–1.29)
Region of residence						
Flanders					(ref.)	(ref.)
Brussels-Capital Region					1.00 (0.88–1.13)	0.98 (0.87–1.12)
Wallonia					1.23 (1.16–1.31)	1.23 (1.16–1.31)
**Squamous cell carcinoma**	**Model 1**	**Model 2**	**Model 3**	**Model 4**	**Model 5**	**Model 6**
Migrant origin						
Belgian	(ref.)	(ref.)	(ref.)	(ref.)	(ref.)	(ref.)
Italian	1.12 (1.00–1.25)	1.13 (1.01–1.27)	0.90 (0.80–1.00)	1.11 (0.99–1.24)	1.02 (0.91–1.14)	0.85 (0.75–0.95)
Turkish	1.17 (0.92–1.49)	1.24 (0.97–1.57)	0.86 (0.68–1.09)	1.06 (0.83–1.35)	1.21 (0.95–1.53)	0.89 (0.70–1.14)
Moroccan	0.68 (0.55–0.84)	0.72 (0.58–0.88)	0.51 (0.41–0.63)	0.59 (0.47–0.72)	0.73 (0.59–0.91)	0.54 (0.43–0.67)
Age	1.09 (1.09–1.09)	1.09 (1.09–1.09)	1.08 (1.07–1.08)	1.09 (1.09–1.09)	1.09 (1.09–1.09)	1.08 (1.08–1.08)
Civil status						
Single		1.25 (1.16–1.34)				1.05 (0.97–1.13)
Divorced		1.44 (1.36–1.53)				1.18 (1.11–1.26)
Widow		1.26 (1.14–1.39)				1.12 (1.02–1.24)
Married		(ref.)				(ref.)
Educational attainment						
Primary or less			3.21 (2.97–3.46)			3.02 (2.80–3.26)
Lower secondary			2.37 (2.20–2.56)			2.28 (2.11–2.46)
Upper secondary			1.74 (1.60–1.89)			1.71 (1.57–1.86)
Tertiary			(ref.)			(ref.)
Missing			3.34 (3.08–3.63)			2.94 (2.70–3.19)
Home ownership						
Tenant				1.78 (1.70–1.86)		1.58 (1.50–1.66)
Owner				(ref.)		(ref.)
Missing				1.71 (1.57–1.87)		1.38 (1.26–1.50)
Region of residence						
Flanders					(ref.)	(ref.)
Brussels-Capital Region					0.89 (0.81–0.98)	0.86 (0.78–0.94)
Wallonia					1.22 (1.17–1.28)	1.22 (1.17–1.28)
**Adenocarcinoma**	**Model 1**	**Model 2**	**Model 3**	**Model 4**	**Model 5**	**Model 6**
Migrant origin						
Belgian	(ref.)	(ref.)	(ref.)	(ref.)	(ref.)	(ref.)
Italian	1.25 (1.13–1.38)	1.26 (1.14–1.39)	1.11 (1.00–1.23)	1.24 (1.12–1.38)	1.12 (1.01–1.24)	1.01 (0.91–1.12)
Turkish	0.96 (0.76–1.22)	0.99 (0.78–1.26)	0.81 (0.64–1.03)	0.90 (0.71–1.14)	0.91 (0.72–1.15)	0.76 (0.60–0.96)
Moroccan	1.04 (0.89–1.21)	1.06 (0.91–1.24)	0.88 (0.75–1.03)	0.91 (0.78–1.07)	0.93 (0.79–1.09)	0.76 (0.64–0.89)
Age	1.05 (1.05–1.06)	1.05 (1.05–1.06)	1.05 (1.04–1.06)	1.05 (1.05–1.06)	1.05 (1.05–1.06)	1.05 (1.05–1.05)
Civil status						
Single		0.98 (0.91–1.05)				0.85 (0.79–0.91)
Divorced		1.36 (1.28–1.44)				1.13 (1.07–1.20)
Widow		1.16 (1.05–1.29)				1.06 (0.96–1.18)
Married		(ref.)				(ref.)
Educational attainment						
Primary or less			1.79 (1.68–1.90)			1.73 (1.62–1.84)
Lower secondary			1.57 (1.48–1.66)			1.53 (1.44–1.62)
Upper secondary			1.35 (1.27–1.44)			1.34 (1.26–1.43)
Tertiary			(ref.)			(ref.)
Missing			1.88 (1.75–2.01)			1.72 (1.60–1.85)
Home ownership						
Tenant				1.69 (1.62–1.76)		1.55 (1.48–1.62)
Owner				(ref.)		(ref.)
Missing				1.41 (1.29–1.53)		1.24 (1.13–1.36)
Region of residence						
Flanders					(ref.)	(ref.)
Brussels-Capital Region					1.33 (1.23–1.43)	1.25 (1.16–1.35)
Wallonia					1.23 (1.18–1.28)	1.22 (1.17–1.27)
**Large-cell undifferentiated carcinoma**	**Model 1**	**Model 2**	**Model 3**	**Model 4**	**Model 5**	**Model 6**
Migrant origin						
Belgian	(ref.)	(ref.)	(ref.)	(ref.)	(ref.)	(ref.)
Italian	0.60 (0.41–0.89)	0.61 (0.42–0.90)	0.51 (0.35–0.75)	0.60 (0.41–0.89)	0.84 (0.56–1.24)	0.73 (0.49–1.08)
Turkish	0.65 (0.29–1.46)	0.68 (0.31–1.53)	0.52 (0.23–1.15)	0.60 (0.27–1.35)	0.74 (0.33–1.65)	0.60 (0.27–1.33)
Moroccan	0.69 (0.41–1.16)	0.71 (0.42–1.21)	0.56 (0.33–0.96)	0.61 (0.36–1.03)	0.90 (0.53–1.54)	0.72 (0.42–1.24)
Age	1.09 (1.08–1.10)	1.09 (1.08–1.10)	1.08 (1.07–1.09)	1.09 (1.08–1.10)	1.09 (1.08–1.10)	1.08 (1.07–1.09)
Civil status						
Single		1.11 (0.92–1.35)				1.00 (0.82–1.21)
Divorced		1.44 (1.23–1.68)				1.31 (1.11–1.53)
Widow		1.37 (1.08–1.75)				1.28 (1.01–1.63)
Married		(ref.)				(ref.)
Educational attainment						
Primary or less			2.54 (2.12–3.03)			2.24 (1.87–2.68)
Lower secondary			1.90 (1.59–2.27)			1.78 (1.49–2.13)
Upper secondary			1.42 (1.17–1.73)			1.33 (1.10–1.63)
Tertiary			(ref.)			(ref.)
Missing			2.30 (1.88–2.81)			2.08 (1.70–2.56)
Home ownership						
Tenant				1.62 (1.43–1.82)		1.51 (1.33–1.71)
Owner				(ref.)		(ref.)
Missing				1.44 (1.14–1.82)		1.30 (1.02–1.65)
Region of residence						
Flanders					(ref.)	(ref.)
Brussels-Capital Region					0.46 (0.35–0.62)	0.44 (0.33–0.59)
Wallonia					0.57 (0.50–0.65)	0.57 (0.50–0.65)
**Other/unknown**	**Model 1**	**Model 2**	**Model 3**	**Model 4**	**Model 5**	**Model 6**
Migrant origin						
Belgian	(ref.)	(ref.)	(ref.)	(ref.)	(ref.)	(ref.)
Italian	0.85 (0.69–1.06)	0.87 (0.70–1.08)	0.70 (0.57–0.87)	0.85 (0.68–1.05)	0.75 (0.60–0.93)	0.65 (0.53–0.81)
Turkish	0.96 (0.63–1.48)	1.03 (0.67–1.59)	0.73 (0.47–1.12)	0.87 (0.56–1.33)	1.01 (0.66–1.56)	0.80 (0.52–1.23)
Moroccan	0.56 (0.38–0.81)	0.59 (0.40–0.86)	0.42 (0.29–0.62)	0.48 (0.33–0.70)	0.63 (0.43–0.93)	0.49 (0.34–0.73)
Age	1.09 (1.08–1.09)	1.09 (1.09–1.09)	1.08 (1.07–1.08)	1.09 (1.08–1.09)	1.09 (1.08–1.09)	1.08 (1.08–1.09)
Civil status						
Single		1.20 (1.06–1.35)				1.02 (0.90–1.15)
Divorced		1.72 (1.57–1.89)				1.42 (1.29–1.57)
Widow		1.21 (1.03–1.43)				1.09 (0.92–1.29)
Married		(ref.)				(ref.)
Educational attainment						
Primary or less			2.50 (2.23–2.82)			2.36 (2.10–2.65)
Lower secondary			1.92 (1.71–2.16)			1.84 (1.63–2.06)
Upper secondary			1.42 (1.26–1.62)			1.40 (1.23–1.59)
Tertiary			(ref.)			(ref.)
Missing			2.65 (2.34–3.01)			2.26 (1.98–2.58)
Home ownership						
Tenant				1.75 (1.62–1.89)		1.54 (1.42–1.66)
Owner				(ref.)		(ref.)
Missing				1.89 (1.65–2.16)		1.54 (1.33–1.77)
Region of residence						
Flanders					(ref.)	(ref.)
Brussels-Capital Region					0.80 (0.68–0.95)	0.75 (0.63–0.88)
Wallonia					1.33 (1.24–1.42)	1.31 (1.22–1.41)

Model 1 is adjusted for age at start of follow-up; Model 2 is adjusted for age at start of follow-up and civil status; Model 3 is adjusted for age at start of follow-up and educational attainment; Model 4 is adjusted for age at start of follow-up and home ownership; Model 5 is adjusted for age at start of follow-up and region of residence; Model 6 is adjusted for all variables of interest. Number of observations = 1,876,628

For overall lung cancer, men from Moroccan descent consistently had a lower incidence risk compared with native Belgians, no matter which indicators were controlled for. Yet, the advantage was largest when adjusting for the indicators of SEP, in particular for educational attainment. For example, in the fully-adjusted model, their overall lung cancer rate was 37% lower compared with native Belgians (IRR: 0.63; 95% CI: 0.56–0.70). This persisting incidence advantage was also observed for SCLC, SCC and lung cancers with unknown/other histology whereas for both ADC and LCLC this advantage was less clear, only in one of the six models.

Migrant men from Turkish descent had 18% lower overall lung cancer incidence (IRR: 0.82; 95% CI: 0.71–0.95) compared with native Belgian men, but only when adjusting for educational attainment (models 3 and 6). Histology-specific, their incidence risk was not statistically different from that of native Belgian men, with only one exception: after taking into account all of the confounders (model 6), they had a 24% lower risk to be diagnosed with ADC (IRR: 0.76; 95% CI: 0.60–0.96) compared with native Belgians.

For men with an Italian background, the pattern was very histology-specific. Their overall lung cancer incidence risk was higher compared with native Belgian men. Yet, when accounting for educational attainment (model 3 and 6), this pattern reversed showing in the final model a lung cancer incidence rate that was 15% lower compared to native Belgians (IRR: 0.85; 95% CI: 0.80–0.91). This change of direction after adding education to the model also appeared for SCC incidence. Educational attainment also seemed to play an important role in SCLC and lung cancer incidence with unknown/other histology. Italian men showed similar SCLC incidence risks as native Belgians, but by adding education and region of residence, this association had turned into an advantage with in the final model an IRR of 0.70 (95% CI: 0.58–0.83) compared to native Belgian men. On the other hand, Italians showed elevated ADC incidence rates compared with native Belgians in models 1 to 5. Only in the full-adjusted model, this association was no longer significant. Finally, the incidence pattern of LCLC was lower among Italian migrants compared to native Belgians, yet this advantage was explained by adding region of residence to the models (5 and 6).

For all four histological subtypes, all confounders showed similar associations, with higher relative incidence with increasing age, not being married, and among the deprived categories (i.e. lower education and tenants). Generally living in Wallonia was associated with a higher incidence risk compared with living in Flanders, except for LCLC for which the association was reversed. The incidence risk of living in Brussels as compared to living in Flanders varied by histological subtype.

## Discussion and conclusion

### Methodological reflections

This study was the first one in Belgium and Europe to precisely assess lung cancer incidence rates by histological subtype and migrant group (country of birth), as well as the extent by which these differences were associated with sociodemographic and socioeconomic characteristics. Belgium represents a unique setting for such a study given the large share of migrants and the availability of a nationwide cancer registry.

We used individually-linked nationwide data combining administrative information on sociodemographics, socioeconomics, mortality and emigration with clinical and pathological information on cancer occurrence during a 10-year follow-up period. Previous research proved it to be important to examine histological subtypes separately as their association with risk factors (e.g. smoking) is different [38], which was also observed in our results. In addition, migrants are a very heterogeneous group in terms of exposures, language, cultural and religious background, social situation, health behaviour and health literacy [47], which are all related to health outcomes and make it therefore essential to study migrant differentials by country of origin [48]. For this study, we conducted a retrospective register-based cohort study, based on the legal population of Belgian men, and FG migrant men from Italian, Turkish and Moroccan descent residing in Belgium for more than 10 years, between the ages of 50–74 years at the start of the follow-up. We followed this cohort 10 years over time until they got diagnosed with lung cancer; disappeared from the cohort because of reaching the age of 75 years, death or emigration; or until the end of follow-up. Such a cohort study design has been shown to be apt to study differences in incidence [49]. The downside of this design is that we made use of a semi-closed cohort, in which no new entries were allowed. Another challenge associated with this study design is selection bias in the composition of the cohort, as well as in the population that is lost to follow-up [49]. We did not consider the first one to be an issue since we disposed of population-wide data. Bias in lost to follow-up would be problematic if migrants who would feel ill -but who would not be diagnosed yet- would return to their home country. Yet, previous research has shown that the salmon-bias hypothesis -migrants leaving the host country due to illness or imminent mortality- was not observed in Belgium, definitely not among migrants being longer than 10 years in Belgium [12].

In addition to documenting cancer incidence patterns by country of origin, we also assessed the role of different indicators of the social situation into this. As migrants often are situated in the more disadvantaged strata, it is important to study whether SEP can explain the observed differences, and more specifically which indicators of SEP, as they represent different forms of disadvantages during different life stages [9]. It is essential to study the contributions of both migration, SEP and sociodemographic variables as they are all associated with disease [43, 50]. Also, to provide clues on policy priorities it is essential to disentangle these factors.

Another merit of this linked dataset was the 10-year observation period for lung cancer incidence, based on cancer registration information covering more than 95% of the Belgian population, together with detailed tumour information [51]. This allowed us to look into stage at diagnosis, which is an important marker of survival [42]. Yet, about one in five of the overall cancers had missing information on this variable, with variation by histological subtype and migrant origin. Stage at diagnosis was in particular likely to be missing for new diagnosed SCLC as well as for Italian migrant men. This may suggest differences in the process of diagnosing between the different origin groups [42] and perhaps also histological subtypes, a topic that definitely needs further consideration.

The linked database however did not contain information on individual-level smoking characteristics such as smoking history, intensity of smoking, second-hand smoke, smoking behaviour of partner, type of tobacco product, all key indicators in lung cancer onset [36]. Additionally, no data were available on other possible carcinogens such as air pollution, radon exposure, environmental exposures, occupational exposure to certain chemicals or history of lung disease and genetic factors. Including such variables would be helpful to enhance the knowledge on the reasons behind the differences in histology-specific lung cancer incidence rates. Because of this lack of individual-level risk factors, we need to be prudent in the interpretation of the mainly descriptive results. Additionally, in this paper we took the native Belgians as a reference to compare the incidence patterns of migrants with. However, comparing with incidence patterns in the home countries could be informative as well but was not possible due to the lack of nationwide cancer registries in the home countries where cancer registries operate more as regional initiatives [52, 53]. Though, taking a glimpse at the most recent overall lung cancer incidence figures on the Globocan website for Belgian, Italian and Turkish men aged 50–74 years suggested lower lung cancer incidence among Italian men compared with Belgian men, but higher incidence among Turkish men [54]. In contrast, among the migrant population in Belgium, we did not observe these differences in overall lung cancer incidence. Yet, we need to be cautious with comparing to these data as the Globocan data did not report for the same follow-up period, but only for 4 years; as both studies used a different standard population: the Globocan used the World Standard Population whereas we used the total Belgian population at the start of the follow-up period; as there were no data available for Morocco; and as the data for Italy came from 33 different regional registries and for Turkey from four regional registries. Next, we should be aware of the fact that taking the opportunity to analyse lung cancer incidence patterns in detail by breaking down the histological subtype and migrant origin caused low numbers in some groups. Caution in the interpretation of some of these figures is thus definitely needed. Finally, another caveat of this study was the fact that undocumented migrants were not included in this study since we made use of linked administrative data on official residents of Belgium.

### Reflections on the main findings of the study

We recall the three research questions when discussing the main findings of this study. The first issue we wanted to find out was *whether overall and histology-specific lung cancer rates differed between migrant and native Belgian men*. The answer was yes, we clearly observed some differences in incidence rates by country of origin. Moroccan men seemed to be the most advantaged group in terms of lung cancer incidence. Both in absolute and relative terms, Moroccan men had consistently lower overall and histology-specific incidence rates as compared with native Belgian men, albeit less clear for ADC and LCLC. The fact that the advantage was very outspoken for SCLC and SCC but less for ADC, points to the fact that the observed differences may be related to smoking, as the first types are strongly related to tobacco smoking and ADC only to a lesser extent [32–34, 36, 40]. This suggests that Moroccan men probably consume less often tobacco or in lower amounts [20, 27]. In addition, their religious beliefs which involve alcohol abstinence may be a protective factor that offsets the negative consequences of smoking [2]. Yet even if it is very likely that they share the same religious practices, Turkish men did not differ significantly with Belgian men for SCLC and SCC incidence, and only in the fully adjusted model they had lower ADC incidence. This observation was also reflected in Belgian mortality figures [20]. Also when comparing World Health Organization data on the prevalence of current tobacco smoking in adults in Belgium and Turkey, the percentages were quite similar with 27.6% smokers in Turkey and 28.3% in Belgium in 2016 [55]. Similarly, in France and Germany, a convergence of lung cancer incidence rates of Turkish migrants to that of the natives was observed, probably due to the adaptation of a more western lifestyle when it comes to levels of tobacco consumption [1, 15, 29]. On the contrary, Italian men had no advantageous lung cancer incidence pattern. They showed higher ADC incidence compared with Belgian men, both in absolute and relative terms, as well as a higher overall lung cancer incidence in relative terms. As mentioned before, ADC is the lung cancer subtype which is the least strong associated with smoking, and for which the full aetiology is not yet known [32]. A possible explanation for the elevated ADC incidence pattern among Italian men may be their labour history and more specifically the exposure to carcinogenic radon while working in the mines [36]. The observation that Italian migrants did have similar smoking-related incidence rates than Belgian men contrasts the existing smoking patterns in Italy, which are lower (23.8) than in Belgium [55]. This may suggest that Italian men may have adapted their smoking habits to the Belgian lifestyle.

Secondly, we wanted to examine whether there were *differences in tumour characteristics between the different migrant groups and native Belgian men, taking the histological subtype into account*. A first observation in this regard was that within our study population of Belgian male residents, ADC was the most common histological type of lung cancer, followed by SCC and SCLC. This finding may be related to the chemical composition of cigarettes [33, 39, 40, 56]. More specifically, filter cigarettes may be associated with deeper inhalations of smoke to satisfy the need for nicotine, which affects the more peripheral regions in the lung where ADC develop [36, 39, 40, 56]. The incidence of ADC may also be related to non-tobacco carcinogens such as environmental pollution that are inhaled more deeply and therefore also reach the peripheral regions of the lung [40]. Additionally, in line with previous findings [25], this study showed that SCLC was more often diagnosed in later stages than the other histological subtypes, which may have implications for the disease prognosis [42], which is already worst in terms of survival for SCLC [57]. In some cases, migrants were diagnosed at later stages as well, e.g. Turkish men with SCC, whereas in other cases native Belgians were diagnosed later, e.g. for SCLC. Nonetheless, differences in delayed diagnosis, but also differences in missing stage at diagnosis, or even differences in smoking prevalence, may reflect differences in health care access and utilization [42].

The third objective of this study was to assess whether *accounting for sociodemographic and socioeconomic variables altered the association between country of birth and histology-specific lung cancer incidence*. Adding these variables to the relative models did alter the association. In particular educational attainment proved to be an important indicator related to lung cancer incidence differences. The strong association between education and lung cancer, has also been shown for lung cancer mortality [58]. This association reflects differences in resources that can be used to maximize one’s health [59] such as health literacy; being receptive to prevention messages; being able to change health behaviour; and making proper use of the health system [60, 61]. A striking example to illustrate this was SCC incidence among Italian men. In the models adjusted for age and civil status only, Italian men had an elevated risk for SCC incidence compared with native Belgian men, however adjusting for educational attainment reversed the association to their advantage. As SCC incidence is related to smoking behaviour, the association with education can be explained by the smoking epidemic [62]. Belgium has gone through all four phases of the smoking epidemic, which means that knowledge about the negative impact of smoking on health is widespread and that smoking is more common among the lower socioeconomic strata [58, 62]. These results proved the importance of adding other variables of interest into the models, in particular indicators of SEP, that clearly played a role in the observed lung cancer incidence differences between migrants and the Belgian host population. However, even after accounting for the social situation, differences in lung cancer incidence by origin group remained, which suggests there must be other factors at play as well.

### Implications and conclusion

We deemed this study to be an interesting step in unravelling the puzzle of lung cancer incidence differences by migrant group and SEP. Bearing in mind the delayed diagnosis among certain groups, we need to ensure that access to health care is spread equally across cultural boundaries. Considering the increasing ages of the labour migrant population in Belgium, urgent actions are needed in this regard as well as the continuously monitoring of their health status and health needs. In addition, it is important to investigate risk factor patterns (i.e. smoking patterns) among migrant groups in Belgium [3] to see which groups need to be tackled in terms of primary prevention. To date, no such figures are available for Belgium, yet, a study in France showed converging rates of smoking patterns among migrants after arrival in the host country [63].The striking results for elevated ADC incidence among Italian migrants need to be further examined in order to identify associated risk factors. The full aetiology for adenocarcinoma is still unknown [32], yet the higher incidence among Italian men could point to differences in occupational exposures, e.g. to carcinogenic radon while working in the mines [36]. Future research is also needed to continue on this path of trying to identify the missing pieces. It would be worthwhile to look into histology-specific lung cancer incidence patterns among various migrant and/or social groups while controlling for other relevant factors that may impact the incidence patterns. Suggested factors that may play a role are for instance air pollution [36, 40], occupation [37, 64] or diet and body mass index [36, 56, 65–67]. Moreover, future studies should repeat this exercise by comparing migrant women’s histology-specific lung cancer incidence patterns with those of native Belgian women. As most migrant women did not work in similar industries as migrant men did, such studies might enhance the knowledge on the aetiology of ADC and other histologies. Besides, such studies are needed as we cannot ignore the fact that overall and histology-specific lung cancer incidence (except for LCLC [25]) is increasing among women [32].

## Data Availability

Data are from a census-cancer registry-linked mortality follow-up study and cannot be made available due to privacy issues. Researchers can gain full access to the data by submitting an application to the Privacy Commission Belgium. In order to get permission to use data from the Belgian population register linked to census data an authorization request (in Dutch or French) needs to be submitted to the Belgian Privacy Commission. The authorization request includes an application form and additional forms regarding data security. The necessary forms for the authorization request can be ﻿downloaded from the Privacy Commission website (www.privacycommission.be). ﻿Next to information on the applicant and a list of requested data, the authorization request should specify why the data from the population register are necessary, for which time span data will be stored, and who will have access to the data.

## Appendix

**Table 5:** ICD-O-3 codes defining the histological subtypes

Histological subtype	ICD-O-3 code
Small-cell lung carcinoma	8041–8045,8246
Squamous cell carcinoma	8050–8078, 8083–8084
Adenocarcinoma	8140, 8211, 8230–8231, 8250–8260, 8323, 8480–8490, 8550–8551, 8570–8574, 8576
Large-cell undifferentiated carcinoma	8010–8012, 8014–8031, 8035, 8310
Other (other and unspecified)	8013,8032-8034,8036-8040,8046-8049,8079-8082,8085-8139,8141-8210,8212-8229,8232-8245,8247-8249,8261-8309,8311-8322,8324-8479,8491-8549,8552-8569,8575 8577–9999, 8000–8005

## References

[1] Arnold M, Razum O, Coebergh J-WW (2010). Cancer risk diversity in non-western migrants to Europe: an overview of the literature. Eur J Cancer.

[2] Wallace M, Kulu H (2014). Low immigrant mortality in England and Wales: a data artefact?. Soc Sci Med.

[3] Kaucher S, Kajüter H, Becher H, Winkler V (2018). Cancer incidence and mortality among ethnic German migrants from the former Soviet Union. Front Oncol.

[4] Winkler V, Holleczek B, Stegmaier C, Becher H (2014). Cancer incidence in ethnic German migrants from the former Soviet Union in comparison to the host population. Cancer Epidemiol.

[5] Spallek J, Zeeb H, Razum O (2011). What do we have to know from migrants’ past exposures to understand their health status? A life course approach. Emerg Themes Epidemiol.

[6] Parkin DM, Khlat M (1996). Studies of cancer in migrants: rationale and methodology. Eur J Cancer.

[7] Llácer A, Zunzunegui MV, Del Amo J, Mazarrasa L, Bolůmar F (2007). The contribution of a gender perspective to the understanding of migrants’ health. J Epidemiol Community Health.

[8] Gadeyne S. The ultimate inequality: socio-economic differences in all-cause and cause-specific mortality in Belgium in the first part of the 1990s. Brussels: Centrum voor Bevolkings-en Gezinsstudie-CBGS; 2006.

[9] Menvielle G, Kunst A (2008). Social inequalities in cancer incidence and cancer survival: lessons from Danish studies. Eur J Cancer.

[10] Spallek J, Arnold M, Razum O, Juel K, Rey G, Deboosere P, Mackenbach JP, Kunst AE (2012). Cancer mortality patterns among Turkish immigrants in four European countries and in Turkey. Eur J Epidemiol.

[11] Van Hemelrijck WMJ, de Valk HAG, Vandenheede H (2017). Cancer mortality by migrant background in Belgium during the 2000s: patterns and social determinants. Cancer Treat Res Commun.

[12] Vandenheede H, Willaert D, De Grande H, Simoens S, Vanroelen C (2015). Mortality in adult immigrants in the 2000s in Belgium: a test of the “healthy migrant” and the “migration-as-rapid-health-transition” hypotheses. Tropical Med Int Health.

[13] Arnold M, Aarts MJ, Siesling S, Van Der Aa M, Visser O, Coebergh JW (2013). Diverging breast and stomach cancer incidence and survival in migrants in The Netherlands, 1996-2009. Acta Oncol (Madr).

[14] Williams G, Mans DRA, Garssen J, Visser O, Kramer D, Kunst AE (2013). Cancer incidence and mortality of Surinamese migrants in the Netherlands: in-between Surinamese and Dutch levels?. Cancer Causes Control Control.

[15] Spallek J, Arnold M, Hentschel S, Razum O (2009). Cancer incidence rate ratios of Turkish immigrants in Hamburg, Germany: a registry based study. Cancer Epidemiol.

[16] Norredam M, Krasnik A, Pipper C, Keiding N (2007). Cancer incidence among 1st generation migrants compared to native Danes--a retrospective cohort study. Eur J Cancer.

[17] Marmot MG, Adelstein AM, Bulusu L (1984). Lessons from the study of immigrant mortality. Lancet..

[18] Norredam M, Nielsen SS, Krasnik A (2010). Migrants’ utilization of somatic healthcare services in Europe - a systematic review. Eur J Pub Health.

[19] Arnold M, Aarts MJ, Van Der Aa M, Visser O, Coebergh JW (2012). Investigating cervical , oesophageal and colon cancer risk and survival among migrants in The Netherlands. Eur J Pub Health.

[20] Deboosere P, Gadeyne S (2005). Adult migrant mortality advantage in Belgium: evidence using census and register data. Popul..

[21] Anson J (2004). The migrant mortality advantage: a 70 month follow-up of the Brussels population. Eur J Popul.

[22] de Valk HAG, Huisman C, Noam KR (2011). Migration patterns and immigrant characteristics in North-Western Europe. Interregional workshop on strengthening capacities to deal with international migration: “Examining development, institutional and policy aspects of migration between Africa, Europe and Latin America and the Caribbean.”.

[23] Bartley M (2004). Health Inequality. An introduction to theories, concepts and methods.

[24] Link BG, Phelan J. Social Conditions as Fundamental Causes of Disease. J Health Soc Behav. 1995;(Extra issue):80–94. 7560851

[25] Belgian Cancer Registry. Cancer Burden in Belgium 2004-2017. Belgian Cancer Registry. Brussels; 2020.

[26] Ferlay J, Colombet M, Soerjomataram I, Dyba T, Randi G, Bettio M, Gavin A, Visser O, Bray F (2018). Cancer incidence and mortality patterns in Europe: estimates for 40 countries and 25 major cancers in 2018. Eur J Cancer.

[27] Razum O, Twardella D (2002). Time travel with Oliver twist-towards an explanation for a paradoxically low mortality among recent immigrants. Trop Med Int Heal.

[28] Ott JJ, Paltiel AM, Winkler V, Becher H (2010). The impact of duration of residence on cause-specific mortality: a cohort study of migrants from the former Soviet Union residing in Israel and Germany. Heal Place.

[29] Mackenbach JP, Bos V, Garssen MJ, Kunst AE (2005). Sterfte onder niet-westerse allochtonen in Nederland. Ned Tijdschr Geneeskd.

[30] Hjerkind KV, Qureshi SA, Møller B, Weiderpass E, Deapen D, Kumar B, Ursin G (2017). Ethnic differences in the incidence of cancer in Norway. Int J Cancer.

[31] Sander, M. Return Migration and the "Healthy Immigrant Effect", SOEPpapers on Multidisciplinary Panel Data Research, No. 60, Deutsches Institut für Wirtschaftsforschung (DIW), Berlin; 2007.

[32] Khuder SA (2001). Effect of cigarette smoking on major histological types of lung cancer : a meta-analysis. Lung Cancer.

[33] IARC (2004). Working group on the evaluation of carcinogenic risks to humans. IARC monographs on the evaluation of carcinogenic risks to humans. Volume 83. Tobacco smoke and involuntary smoking. Vol. 83.

[34] Kim CH, Amy Lee Y-C, Hung RJ, McNallan SR, Cote ML, Lim W-Y (2015). Exposure to secondhand tobacco smoke and lung cancer by histological type: a pooled analysis of the international lung Cancer consortium (ILCCO). Int J Cancer.

[35] Shimizu H, Nagata C, Tsuchiya E, Nakagawa K, Weng S-Y (1994). Risk of lung cancer among cigarette smokers in relation to tumor location. Jpn J Cancer Res.

[36] Dela Cruz CS, Tanoue LT, Matthay RA (2011). Lung Cancer: epidemiology, etiology, and prevention. Clin Chest Med.

[37] De Stefani E, Boffetta P, Ronco AL, Brennan P, Correa P, Deneo-pellegrini H (2005). Squamous and small cell carcinomas of the lung: similarities and differences concerning the role of tobacco smoking. Lung Can.

[38] Kabat GC (1996). Aspects of the epidemiology of lung cancer in smokers and nonsmokers in the United States. Lung Cancer.

[39] Yang CP, Gallager RP, Weiss NS, Band PR, Thomas DB, Russell DA (1989). Differences in incidence rates of cancers of the respiratory tract by anatomic subsite and histologic type : an etiologic implication. J Natl Cancer Inst.

[40] Franceschi S, Bidoli E (1999). The epidemiology of lung cancer. Ann Oncol.

[41] Muscat JE, Wynder EL (1995). Lung cancer pathology in smokers , ex-smokers and never smokers. Cancer Lett.

[42] Cho A, Bin JP, Holleczek B, Becher H, Winkler V (2018). Stage of cancer diagnoses among migrants from the former Soviet Union in comparison to the German population - are diagnoses among migrants delayed?. BMC Public Health.

[43] Maringe C, Mangtani P, Rachet B, Leon DA, Coleman MP, dos Santos Silva I (2013). Cancer incidence in south Asian migrants to England, 1986-2004: unraveling ethnic from socioeconomic differentials. Int J Cancer.

[44] Brierley J, Gospodarowicz MK, Wittekind C (2017). Union for International Cancer Control. TNM classification of malignant tumour.

[45] Norredam M, Agyemang C, Hoejbjerg Hansen OK, Petersen JH, Byberg S, Krasnik A, Kunst AE (2014). Duration of residence and disease occurrence among refugees and family reunited immigrants: test of the “healthy migrant effect” hypothesis. Trop Med Int Heal..

[46] Acevedo-Garcia D, Bates LM, Osypuk TL, McArdle N (2010). The effect of immigrant generation and duration on self-rated health among US adults 2003-2007. Soc Sci Med.

[47] Leonhardt M, Aschenbrenner K, Kreis ME, Lauscher JC (2018). Exploring the characteristics and potential disparities of non-migrant and migrant colorectal cancer patients regarding their satisfaction and subjective perception of care – a cross-sectional study. BMC Health Serv Res.

[48] Boulogne R, Jougla E, Breem Y, Kunst AE, Rey G (2012). Mortality differences between the foreign-born and locally-born population in France (2004-2007). Soc Sci Med.

[49] Grimes DA, Schulz KF (2002). Cohort studies: marching towards outcomes. Lancet..

[50] Cokkinides VE, Bandi P, Siegel RL, Jemal A (2012). Cancer-related risk factors and preventive measures in US Hispanics / Latinos. CA Cancer J Clin.

[51] Henau K, Van Eycken E, Silversmit G, Pukkala E (2015). Regional variation in incidence for smoking and alcohol related cancers in Belgium. Cancer Epidemiol.

[52] Tazi MA, Er-Raki A, Benjaafar N. Cancer incidence in Rabat, Morocco: 2006-2008. Ecancermedicalscience. 2013;7:338. 10.3332/ecancer.2013.338 PMC3737118 23940493

[53] Eser S, Yakut C, Özdemir R, Karakılınç H, Özalan S, Marshall SF (2010). Cancer incidence rates in Turkey in 2006: a detailed registry based estimation. Asian Pacific J Cancer Prev.

[54] Bray F, Ferlay J, Laversanne M, Brewster DH, Gombe Mbalawa C, Kohler B, Piñeros M, Steliarova-Foucher E, Swaminathan R, Antoni S, Soerjomataram I, Forman D (2015). Cancer incidence in five continents: inclusion criteria, highlights from volume X and the global status of cancer registration. Int J Cancer.

[55] World Health Organization. European Health for All Database. 2020. Available from: https://gateway.euro.who.int/en/hfa-explorer/.

[56] Wynder EL, Muscat JE (1993). The changing epidemiology of smoking and lung Cancer histology. Environ Health Perspect.

[57] Campobasso O, Invernizzi B, Musso M, Berrino F (1974). Survival rates of lung cancer according to histological type. Br J Cancer.

[58] Vanthomme K, Vandenheede H, Hagedoorn P, Gadeyne S. Evolution of educational inequalities in site-specific cancer mortality among Belgian men between the 1990s and 2000s using a "fundamental cause" perspective. BMC Cancer. 2017;17(1):470. 10.1186/s12885-017-3461-8 PMC5498997 28679369

[59] Menvielle G, Chastang J, Luce D, Leclerc A (2007). Evolution temporelle des inégalités sociales de mortalité en France entre 1968 et 1996. Etde en fonction du niveau des études par cause de décès. Rev Epidemiol Santé Publique.

[60] Jemal A, Ward E, Anderson RN, Murray T, Thun MJ (2008). Widening of socioeconomic inequalities in U.S. death rates, 1993-2001. PLoS One.

[61] Menvielle G, Leclerc A, Chastang J-F, Luce D (2010). Socioeconomic inequalities in cause specific mortality among older people in France. BMC Public Health.

[62] Lopez AD, Collishaw NE, Piha T (1994). A descriptive model of the cigarette epidemic in developed countries. Tob Control.

[63] Khlat M, Legleye S, Bricard D (2018). Migration-related changes in smoking among non-Western immigrants in France. Eur J Pub Health.

[64] Muscat JE, Stellman SD, Wynder EL (1995). Insulation, Asbestos, smoking habits, and lung Cancer cell types. Am J Ind Med.

[65] Zhu H, Zhang S (2018). Body mass index and lung cancer risk in never smokers : a meta-analysis. BMC Cancer.

[66] Kagohashi K, Satoh H, Kurishima K, Ishikawa H, Ohtsuka M (2006). Body mass index and lung cancer risk in never smokers. Radiol Oncol.

[67] Kabat GC, Wynder EL (2018). Body mass index and lung Cancer risk. Am J Epidemiol.

